# MR-Guided Radiotherapy for Rectal Cancer: Current Perspective on Organ Preservation

**DOI:** 10.3389/fonc.2021.619852

**Published:** 2021-03-30

**Authors:** Luca Boldrini, Martijn Intven, Michael Bassetti, Vincenzo Valentini, Cihan Gani

**Affiliations:** ^1^Unità Operativa Complessa Radioterapia Oncologica, Dipartimento di Diagnostica per Immagini, Radioterapia Oncologica ed Ematologia, Fondazione Policlinico Universitario “A. Gemelli” IRCCS, Roma, Italy; ^2^Istituto di Radiologia, Università Cattolica del Sacro Cuore, Roma, Italy; ^3^Department of Radiation Oncology, University Medical Center Utrecht, Utrecht, Netherlands; ^4^Department of Human Oncology, University of Wisconsin-Madison, Madison, WI, United States; ^5^Department of Radiation Oncology, Eberhard Karls Universität Tübingen, Tübingen, Germany; ^6^German Cancer Consortium (DKTK), Partner Site, Tübingen, Germany

**Keywords:** radiotherapy, rectal cancer, MRI, MR-linac, dose escalation

## Abstract

Online MRI-guided radiotherapy (MRgRT) is one of the most recent technological advances in radiotherapy. MRgRT permits the visualization of tumorous and healthy tissue while the patient is on the treatment table and online daily plan adaptations following the observed anatomical changes. In the context of rectal cancer, online MRgRT is a very promising modality due to the pronounced geographical variability of tumor tissues and the surrounding healthy tissues. This current paper will discuss the possible applications of online MRgRT, in particular, in terms of radiotherapy dose escalation and response prediction in organ preservation approaches for rectal cancer.

## Introduction

Neoadjuvant (chemo)radiotherapy (NCRT) represents the reference standard in the treatment of locally advanced rectal cancer (LARC), primarily aiming to reduce local recurrence rates after surgery (1). MRI with its superior soft-tissue contrast has gained a crucial role in the initial staging and response assessment of rectal cancer and can stratify patients into different prognostic groups with risk-adjusted personalized therapeutic approaches (2, 3).

A promising driver of precision RT in rectal cancer is the recent introduction of linear accelerators with an onboard MR scanner, the MR-Linac. This new treatment machine enables online MRI-guided RT (MRgRT) which opens a new era for an image-guided and online adaptive RT (4). At the time of writing, two commercial 35 solutions are available for clinical use: the MRIdian system by ViewRay (ViewRay Inc, USA), which was first released in 2014 coupling a low tesla scanner (0.35 T) with a triplet of ^60^Co heads and was later replaced by a 6 MV linac, and the Unity system by Elekta (Elekta AB, Sweden), which uses a 1.5 T scanner and a 7 MV linac, released in 2017 (5–7). Despite the low number of active hybrid units, there is a growing interest on the role of this advanced irradiation technique (4, 8).

One of the areas of interest and current research on rectal cancer is the organ preservation approach (9). With the current therapeutic approaches, pathological complete response (pCR) after NCRT for LARC is in the range of 10–20% in most trials. There has been a great interest in developing strategies with tolerable toxicity to increase the number of patients who achieve a complete clinical response and, therefore, could be managed in a non-operative manner in the framework of a “watch and wait” approach (10, 11). These strategies include the intensification of systemic treatment, the prolongation of the interval between neoadjuvant therapies and response assessment or surgery, total neoadjuvant therapy, hyperthermia, and radiotherapy dose escalation (12–16). The latter strategy has been hampered so far by the very limited resolution of cone-beam CT (CBCT)-based images, resulting in large safety margins. Furthermore, response prediction is a field with a need for tailored treatment approaches.

This review aims to present and discuss opportunities with online MRgRT in the treatment of rectal cancer.

## Adaptations for the Changing Anatomy

**Figure 1:**
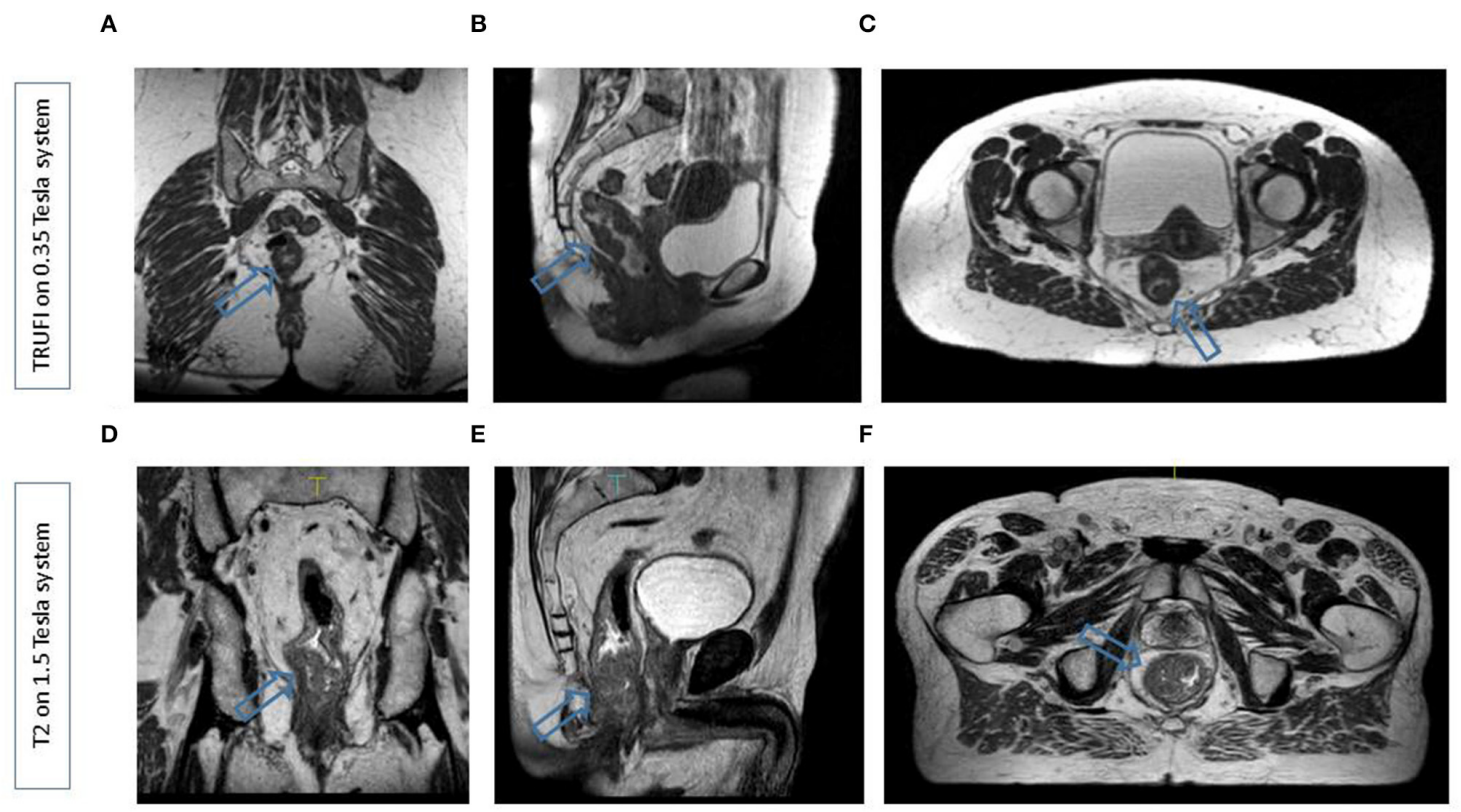
Representative scans from the 0.35 T MR LINAC **(A–C)** in the coronal, sagittal, and transverse planes and the 1.5 T MR LINAC **(D–F)** in the coronal, sagittal, and transverse planes. In both cases, a patient with a distally located rectal tumur was scanned. Arrows indicate the tumur.

Variations in the target position during radiotherapy for rectal cancer are largely due to daily changes in bladder and bowel filling. For the elective target volumes used to treat patients in the neoadjuvant setting, the position variations are most prominent in the mesorectum, specifically in the anterior part of the upper mesorectum, where the position of the mesorectum is dependent on both rectal and bladder filling, and with deformations of up to 7 mm (17–19). Besides interfraction variations due to changes in the filling of the organs in the pelvis, there is also a possibility of tumor regression during the treatment course, which changes the anatomy. On average, rectal tumors can reduce almost 50% in volume during the treatment course (20, 21). These variations are, in most cases, not relevant in the neoadjuvant setting as the gross target volume (GTV) is inside the mesorectum in most cases but becomes very relevant in the setting of dose escalation. Taking into account these uncertainties at the target position, generally large clinical target volume (CTV) to planning target volume (PTV) margins are used around the target volumes of up to even 2.3 cm, which leads to a considerable burden on the healthy tissues (18, 19). Different adaptive strategies have been proposed to reduce the need for large margins in the neoadjuvant treatment setting. One of the most promising techniques is the library of plans (LOP) strategy (22). In this strategy, the CTV from a single planning CT is contracted and expanded based on population variation statistics, and multiple radiation treatment plans are generated based on different CTVs. For each fraction, the plan is chosen with the CTV that best matches the actual volume as visualized on the localization CBCT. Applying the LOP strategy allows reductions of, on average, 15% in the PTV compared to conventional treatment, but the daily selection of the appropriate plan can be challenging due to poor CBCT image quality (20, 21). Furthermore, while this approach is useful for an adequate coverage of the mesorectum, it is no longer a reliable tool for dose escalation of the tumor itself. With MRgRT, it is possible to adapt daily treatment plans based on MRI-visualized anatomy. The superior soft-tissue contrast of MRI compared to CBCT gives the opportunity to not only see the mesorectum and organs at risk but also to visualize the primary tumor and pathological lymph nodes during each fraction. Figure 1 shows representative scans from the 0.35 T and 1.5 T MR-Linac. Based on this daily visualized anatomy, different adaptive treatment strategies can be chosen from a simple translation of the treatment fields to full online replanning (7). The radiotherapy dose escalation strategy takes around 50 min for each treatment fraction and allows the use of smaller CTV to PTV margins of 4–6 mm (23). Reduced margins and daily adaptation of treatment fields lead to a reduced spread of the dose in the surrounding healthy structures, such as the surrounding uninvolved rectal wall, the small bowel, the bladder, and the anal sphincter, potentially resulting in less radiotherapy-related short- and long-time side effects. This is particularly important for the expanding group of patients who are treated with watchful waiting strategies, as, for these patients, a treatment with limited toxicity and a satisfactory anorectal function after (chemo)radiotherapy is of utmost importance (24).

## Dose Escalation

In order to increase the number of patients eligible for organ preservation strategies, innovative and novel treatment protocols to maximize complete response rates are needed. This can be achieved by increasing the radiotherapy dose to the primary tumor as shown in the dose-response curve presented by Appelt et al. (25). This dose-response curve was constructed based on studies that delivered a brachytherapy boost after external beam radiotherapy for locally advanced rectal cancer. Moreover, a systemic review by Burbach et al. showed a potential effect of external beam radiotherapy dose escalation. At the same time, two recent prospective randomized trials did show an increased tumor response with external beam dose escalation, but not in terms of pathological complete response or sustained clinical complete response (26, 27). An explanation for these negative results can at least partly be seen in the limitations of CBCT-based dose escalation, in particular, limited target coverage. Large safety margins had to be used because of the aforementioned poor target, and organ-at-risk visibility with CBCT imaging and organ-at-risk constraints resulted in reduced coverage of the tumor in many cases (28). Another aspect in both the clinical trials was the high complete rate observed in the standard arm underlining the critical need for parameters that identify patients who are unlikely to benefit from dose escalation since they already have a very favorable phenotype. More precise delivery of the external beam irradiation with online MR guidance dose can probably solve the issue of target volume coverage as tumors can be visualized with MRI immediately before and during dose delivery. This solution has a clear advantage of online adaptive MRgRT over “offline” adaptive strategies with pre-defined time points for adaptation (29). Besides, by daily online replanning, the margins needed can be minimized and treatment volumes for dose escalation will be smaller, potentially facilitating dose escalation beyond the biologically effective dose of ~65–70 Gy used in the recently published dose escalation studies (26, 27). This is supported by a recent radiotherapy planning study by Bonomo et al. based on sequential MRI scans, showing that an online adaptive boost strategy results in lower doses to the rectum and the anal canal (30). MR-guided dose escalation strategies are currently under development, and the organ preservation potential of these new schemes will be tested in innovative trials. While the safety of extreme dose escalation under MRI guidance needs clinical proving, the experience in prostate cancer suggests that the rectum can tolerate a high dose localized to a small volume (31). MR-guided dose escalation may also help facilitate an R0 resection in challenging surgical cases. Rectal tumors with threatened mesorectal margins, pelvic sidewall invasion, or iliac lymph node involvement are at high risk for incurable local recurrences. Radiation boost can be used to decrease the risk of positive surgical margin in these cases or to eradicate tumor cells in lymph nodes that are not routinely resected.

## Future Perspectives: Precision RT With the Inclusion of Prediction Models and Functional Imaging

Besides the intuitive approach of using anatomical MR sequences for the adaption of treatment plans following the anatomy of the day, there has been a great interest in using functional imaging data and advanced image analysis for precision radiotherapy of rectal cancer (32, 33). Data from the literature supports various hypotheses on how diffusion-weighted imaging in particular might be a very useful tool. First, it has been shown that early changes in the apparent diffusion coefficient (ADC) can predict response to radiochemotherapy more accurately than early changes in tumor volume (34). Interestingly, the predictive value of early changes in ADC, for instance from baseline to week two of treatment, was superior to baseline ADC values, likely reflecting biological properties of the tumor. As described earlier, adequate patient selection is a key component of dose escalation strategies. A considerable number of patients can achieve a complete response without dose escalation and will have no benefit from dose escalation. Therefore, selecting patients for dose escalation based on changes in functional imaging data is a very interesting approach. With MR-linac hybrid devices, it is possible to acquire these data “in one go,” while the patient is on the table and being treated. Moving one step beyond, one could also envision defining tumor subvolumes that have a high likelihood of harboring residual tumor cells and use these volumes for dose-escalated treatment as supported by a study by Shaverdian et al. (35). Furthermore, the large amount of imaging data that is acquired during the course of the treatment can be used to generate, optimize, and validate prediction models in the context of quantitative imaging data and radiomic analysis (36, 37).

## Data Availability Statement

The original contributions presented in the study are included in the article/supplementary material, further inquiries can be directed to the corresponding author/s.

## Author Contributions

All authors were involved in the writing of this perspective paper. CG and LB provided the figure.

## Conflict of Interest

LB has active research agreements with ViewRay Inc and has received speaker honoraria for scientific presentations. CG: University Hospital Tübingen receives financial and technical support, including costs for travels (CG) and symposia from Elekta AB (Stockholm, Sweden), under a research agreement.

The remaining authors declare that the research was conducted in the absence of any commercial or financial relationships that could be construed as a potential conflict of interest.
